# Lipid-Based Nanostructures for the Delivery of Natural Antimicrobials

**DOI:** 10.3390/molecules26123587

**Published:** 2021-06-11

**Authors:** Cristian Mauricio Barreto Pinilla, Nathalie Almeida Lopes, Adriano Brandelli

**Affiliations:** 1 Laboratory of Applied Microbiology and Biochemistry, Institute of Food Science and Technology (ICTA), Federal University of Rio Grande do Sul, Porto Alegre 91501-970, Brazil; christian_mao@hotmail.com (C.M.B.P.); nathalie.lopes@hotmail.com (N.A.L.); 2 Center of Nanoscience and Nanotechnology (CNANO), Federal University of Rio Grande do Sul, Porto Alegre 91501-970, Brazil

**Keywords:** natural antimicrobials, microbial safety, nanostructures, phospholipids, liposomes

## Abstract

Encapsulation can be a suitable strategy to protect natural antimicrobial substances against some harsh conditions of processing and storage and to provide efficient formulations for antimicrobial delivery. Lipid-based nanostructures, including liposomes, solid lipid nanoparticles (SLNs), and nanostructured lipid nanocarriers (NLCs), are valuable systems for the delivery and controlled release of natural antimicrobial substances. These nanostructures have been used as carriers for bacteriocins and other antimicrobial peptides, antimicrobial enzymes, essential oils, and antimicrobial phytochemicals. Most studies are conducted with liposomes, although the potential of SLNs and NLCs as antimicrobial nanocarriers is not yet fully established. Some studies reveal that lipid-based formulations can be used for co-encapsulation of natural antimicrobials, improving their potential to control microbial pathogens.

## 1. Introduction

The control of undesirable spoilage and/or pathogenic microorganisms has been a constant challenge for diverse fields, including medicine, agriculture and the food and pharmaceutical industries. The occurrence of emerging pathogens and the raising of resistance to conventional antimicrobials makes the search for innovative antimicrobial systems to combat undesirable microbes a topic of utmost interest [1,2]. In this regard, there is an increased demand for natural substances due to the growing concern about the utilization of chemical preservatives in the food sector and the use of conventional antibiotics in medical practice due to the rise of resistant strains. Antimicrobial substances of natural origin appear to be an interesting alternative, and some natural antimicrobials have been investigated as food preservatives and as potential molecules to combat infections. A diversity of natural antimicrobial compounds derived from plants, animals and microorganisms have been proven to show effectiveness in combating microbial pathogens in vitro, in vivo, and in situ on some food matrices [3,4,5,6].

However, many antimicrobial substances of natural origin are very sensitive to some processing and/or storage conditions. Once exposed to relatively high temperature and pressure, these substances can be easily degraded, and the evaporation of volatile antimicrobials takes place. Undesirable interactions with components of the formulations (e.g., lipids, proteins), or modification by endogenous enzymes can occur, resulting in reduction or even loss of antimicrobial activity [7]. For example, medical uses of antimicrobial peptides are limited by their low bioavailability, which has been associated with their poor stability to proteolysis and hydrolysis, low permeability across barriers, and short shelf-life in the circulatory system [8].

Encapsulation is recognized as a valuable approach in providing the protection and effective delivery of natural antimicrobials [9]. Among the several strategies that have been proposed for the encapsulation of antimicrobial compounds, those that use natural substances as wall materials have a great appeal for use in food and biomedical applications. Furthermore, lipid-based nanostructures have been widely investigated for development of potential carriers for drugs, vitamins, enzymes, pigments and natural antimicrobials. Lipid-based systems have demonstrated significant value as drug delivery platforms in biomedical applications. These delivery systems range from simple oil solutions to complex combinations of lipids, surfactants and co-surfactants [10,11]. Liposomes and solid lipid nanoparticles (SLNs) are two important lipid-based nanostructures widely studied as antimicrobial carriers. In addition, nanoemulsions have proven their efficacy for delivery of some natural antimicrobials such as essential oils, and more recently nanostructured lipid carriers (NLCs) have been introduced as the next generation of the SLNs to overlook the potential limitations of SLNs [11,12,13].

As lipid-based nanostructures have similar characteristics to that of natural cellular components, they can easily interact with cells and tissues. Furthermore, they are non-toxic and biodegradable, and can be administered by oral ingestion. These characteristics have made lipid-based nanostructures very interesting for antimicrobial delivery. The aim of this article is to present a critical discussion on current and prospective applications of lipid-based nanostructures for the delivery of natural antimicrobial substances.

## 2. Importance of Natural Antimicrobials

Microbial growth can trigger food decomposition, leading to nutritional and economic losses as a consequence. Furthermore, the ingestion of food contaminated with pathogenic microorganisms cause several foodborne diseases. In order to reduce these problems, for many years, the food industry has been using chemical preservatives to control the growth of undesirable microorganisms and, thus, prolong the shelf life of food. However, the use of synthetic additives has been associated to negative effects, such as adverse health implications. Furthermore, research on natural products have revealed important advances in the discovery of novel antimicrobial compounds to combat infectious diseases, which are one of the main global causes of morbidity and mortality. In this sense, the use of bio-preservatives meets the tendency for healthier and safer products, being additive free and with minimal processing. This tendency has generated a current and actual demand among consumers, compelling food and pharmaceutical industries to use natural alternatives.

Bioactive compounds have been highly documented in the literature for food preservation, e.g., the biomolecules present in essential oils from plants, herb extracts, peptides and enzymes, as shown in Table 1.

Although the use of natural preservatives has received increasing interest, some negative effects may occur, such as the degradation of antimicrobial agents and interaction with food matrix components, as well as undesirable effects on organoleptic properties [4,25,26]. Nanotechnology is one of the alternatives for delivering natural preservatives in food. These natural additives can be encapsulated as natural preservatives for the inclusion in foods, in general, presenting advantages when compared to the use of free antimicrobials [9]. Recent studies have reported that encapsulated antimicrobials present higher stability and bioactivity, targeted delivery, and controlled release [27], improving efficacy for controlling spoilage and pathogenic microorganisms. Thus, the remarkable potential of bioactive compounds, such as natural antimicrobials incorporated into nanostructures, is a current focus in the food research area, and their application has been developed in several products, for example meat, dairy, bread, juice and others [27,28,29,30,31]. However, it is important to select the appropriate carrier for encapsulation, because the interactions between bioactive compounds and the carrier material may influence the natural antimicrobial agent’s activity [27].

## 3. Lipid-Based Nanostructures

Lipid-based nanocarriers have unique properties related to their physiochemical diversity, biodegradability and biocompatibility. Several types of lipid-based nanostructures were developed including liposomes, solid lipid nanoparticles (SLN) and nanostructured lipid carriers (NLC) and nanoemulsions. The use of such nanostructures can improve the bioavailability of poorly permeable and poorly water-soluble drugs, as well as enhance the delivery of bioactive compounds [32]. Liposomes are closed vesicles composed of amphiphilic lipids that can entrap both hydrophobic and hydrophilic molecules [8]. Nanoemulsions are often classified as either oil-in-water (O/W) or water-in-oil (W/O) dispersions stabilized by a surfactant molecule or interfacial film, whose droplet size ranges from 20 to 600 nm [11]. On the other hand, lipid nanoparticles are classified into two categories: (i) SLNs produced from a single solid lipid species and (ii) NLCs formed from blends of liquid lipids (unsaturated fatty acids) and solid lipids [33]. The ability of the nanostructured system is often associated with the characteristics of the antimicrobial substance, the nanomaterial and their application.

### 3.1. Liposomes

**Figure 1:**
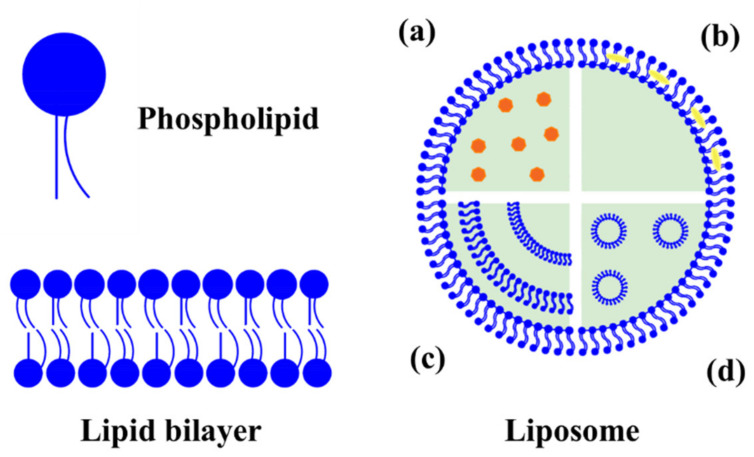
Schematic representation and classification of liposomes: (**a**) hydrophilic molecules encapsulated in water phase of unilamellar vesicle, (**b**) hydrophobic molecules are including in the phospholipid bilayers of unilamellar vesicle, (**c**) multilamellar vesicle, and (**d**) multivesicular vesicle.

One of the most studied lipid-based nanostructures for encapsulation of natural antimicrobials is liposomes. This structure consists of self-assembled closed vesicles with one or more lipid bilayers (generally phospholipids) that separates them from the surrounding water environment [34,35]. In other words, liposomes are spherical structures composed of two layers of lipid molecules that have their nonpolar groups facing towards each other, which have the ability to spontaneously form bilayers in aqueous solutions [36]. Liposomes present an amphiphilic nature, and as a consequence these structures can be used to simultaneously encapsulate compounds of different polarities, such as polar compounds in the aqueous core (Figure 1a), as well as nonpolar compounds in the bilayers of the liposome (Figure 1b). The interaction between lipophilic molecules and phospholipid bilayers is driven by a complex mechanism that includes hydrophobic, polar interactions and hydrogen bonds. When combined with changes in the liposomal permeability and stability, the broad distribution of the compounds in the bilayer and the “weak” nature of some of these interactions could favor the release of encapsulated compounds. Furthermore, liposomes are classified in unilamellar vesicles (ULV) and multilamellar vesicles (MLV), according to the number and structure of lipid bilayer(s) [37], as it can be observed in Figure 1c,d.

Methods of liposome preparation can be divided into conventional and novel techniques. The most conventional methods involve (1) the dissolving of lipid in organic solvent, (2) the evaporation of organic solvent, (3) the dispersion of lipids in aqueous media, and (4) the formation of liposomal suspension [37]. The thin film hydration is the most common method for liposome fabrication, although other techniques have been used, including solvent injection, detergent removal, and homogenization [36]. However, these techniques are not suitable for the encapsulation of sensitive substances because such processes involve the use of organic solvents and detergent residue. Moreover, conventional methods suffer from drawbacks hindering the manufacturing at the industrial scale [35]. To overcome these limitations, other methods have been studied, such as the application of techniques involving heating, dense gases, supercritical fluids and high-pressure homogenization.

The structure of liposomes is appropriate to encapsulate several bioactive compounds, for example, natural antimicrobials. However, these structures are thermodynamically unstable systems, which are prone to aggregate or degrade, resulting in limitations regarding the feasibility of bioactive encapsulation [38,39]. To overcome the limitations related to stability of liposomes, different studies suggest the use of biopolymers as coating materials. In general, biopolymers can help to stabilize particles by modifying the surface of liposomes, through non-covalent or covalent interactions. Thus, the integration of biopolymers (including polysaccharides, proteins, and their derivatives) represents a promising strategy for improving performances of liposomes, making them more protected, stable and consequently applicable [36,39]. Among the biopolymers available to coat liposomes, chitosan is one of the most commonly used [40], besides starch [41], alginate [42] and pectin [43].

### 3.2. Solid Lipid Nanoparticles

**Figure 2:**
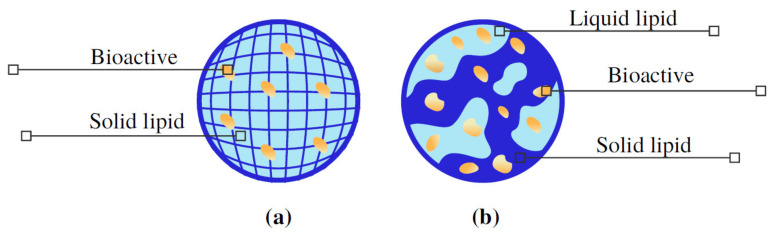
Schematic representation of (**a**) solid lipid nanoparticles (SLNs) and (**b**) nanostructured lipid carriers (NLCs).

The solid lipid nanoparticles (SLNs) were the first generation of lipid nanoparticles obtained using a solid matrix, characterized by the presence of one or more solid lipids (saturated fatty acids). They are interesting lipid-based carriers for a number of reasons, including diameter between 50 and 1000 nm, do not require the use of organic solvents for their production, they have low cost and can be easily scaled up [13]. Thus, SLNs were developed to overcome the disadvantages of other lipid-based nanocarriers. For this, the liquid lipid (oil) was replaced by solid lipid in the emulsion, presenting an organized crystalline structure, allowing the bioactive components to be accommodated within the lipid matrix (Figure 2a). The mobility of encapsulated bioactive compounds in SLNs is controlled by the physical lipid matrix state, which depends on the nanoparticle composition. The prolonged release rate can be associated to viscosity changes of the nanoparticle, as a high amount of lipids results in an increased viscosity of the medium and more rigid solidified nanoparticles. Thus, the ability to move is much lower than those found in liquid droplets of emulsions, because the compounds have a much lower diffusion rate within these solid lipid matrices with high viscosity; consequently, these nanostructures prolong the release period of active agents and protect them against undesirable chemical reactions [44,45].

The dissolution facilitated in the lipid matrix is the most important advantage of encapsulating bioactive components into lipid nanoparticles, such as SLNs. Due to their remarkable benefits, these structures have been used in medicine with application in cosmetics and pharmaceuticals [33,44]. In this regard, the use of SLNs in the food industry appears as a promising alternative for applications in food products, such as carotenoids [46], vitamins [47,48], antioxidants [49] and phytosterols [50], particularly for encapsulating and delivering lipophilic bioactive compounds.

For the development of SLNs it is necessary to know the interactions between the bioactive compounds and the lipid matrix, as well as their chemical and physical properties [33]. The nanoparticles should be obtained by the use of high thermal resistance lipid, with a melting point above 40 °C, to assure that the lipids are solid at room temperature as well as body temperature [32]. The lipidic materials used in the preparation of SLNs include mainly triglycerides, fatty acids, complex glyceride mixtures, and waxes. Moreover, depending on the materials, the addition of a stabilizer such as a surfactant is necessary [13,51].

A number of interesting techniques for obtaining SLNs are available in the literature, including high-pressure homogenization using hot or cold dispersion techniques, solvent emulsification (evaporation and emulsification method), microemulsion, melting dispersion, ultrasonic processing, emulsion technique and solvent injection [44]. However, homogenization and ultrasonic techniques have greater potential for application in the food industry on an industrial scale [33].

Despite all the displayed advantages, the SLNs have some limitations, such as low loading efficiencies and the relatively high water content of the dispersions (70–99.9%). Moreover, in some cases, the expulsion of the bioactive compound incorporated during polymorphic transitions occurs due to a densely packed lipid crystal network, which provides little room for the incorporation of compounds [13,33]. To overcome this negative feature of SLN, a second generation of lipid nanoparticles was produced, referred to as NLCs, described in the sequence.

### 3.3. Nanostructured Lipid Carriers

In order to cover the deficiencies found in SLNs, the second generation of lipid nanoparticles was developed, called nanostructured lipid carriers (NLCs). These nanostructures are formed by a mixture of solid lipids and liquid lipids (unsaturated fatty acids) to improve loading capacity and to inhibit the expulsion of bioactive compounds [32,44]. These nanostructures are systems composed by lipids generally dispersed in water, with a solid lipid to liquid lipid blend preferably in a ratio of 70:30 [33]. Compared to SLNs, the presence of liquid lipids in NLCs prevents the particle from coalescing with the solid matrix, enabling the encapsulation of bioactive ingredients which are better solubilized in the liquid lipids. In addition, the NLCs are formed from blends of spatially different lipid molecules, producing more imperfections in the matrix which allows more encapsulated biocompounds than SLNs, as shown in Figure 2b [13,44,51]. However, despite the presence of liquid lipids, the matrix of this system is solid at body and room temperature in order to maintain the structural and functional properties [33]. Thus, NLCs present advantages over SLNs as described in the previous section, plus the benefits of improving the encapsulation efficiency, higher loading capacity, lower water content and higher bioavailability of encapsulated bioactive compounds.

For the abovementioned reasons, these nanostructures have shown to be a valuable option for application in the food industry. Many research studies have been carried out in order to encapsulate biocompounds into NLCs, for example, vitamin C, vitamin A, curcumin, quercetin, astaxanthin, β-carotene and green tea extract [52,53,54,55,56,57,58].

The key element in the formulation of NLCs is the liquid lipid, including digestible oils. Unsaturated oils such as oleic acid, squalene, vegetable and seed oils (i.e., soybean oil, olive oil, sunflower oil, cottonseed oil and sesame oil) are the most commonly used for the development of these nanostructures [44]. In general, the natural oils have low cost, are environmentally safe and their incorporation enhances the encapsulated components solubility [33]. Additionally, natural oils might contain natural antioxidants and antimicrobial compounds that can improve the protection of the encapsulated compounds.

The techniques for obtaining NLCs are similar to SLNs, producing nanoparticles with different shapes and sizes. There are several methods adopted in order to prepare these lipid nanostructures, however, high-pressure homogenization (hot or cold) is the main method used to obtain these delivery systems. These nanostructures are produced through the combination of lipids (solid and liquid) plus bioactive ingredients in water along with surface active substances that function as emulsifiers (also called surfactants), for example, commercial lecithin, purified phosphatidylcholine, polysorbates, saponins, modified starch, and proteins [45]. Despite some lipids presenting emulsifying properties, the incorporation of additional emulsifiers is often needed to provide stability against nanoparticle aggregation. In conventional emulsions, the emulsifier influences the stability of the dispersions, providing sufficient repulsive forces to prevent flocculation or coalescence of the systems, also influencing the particle size that can be obtained during homogenization. However, in lipid nanoparticles, the addition of emulsifiers presents a fundamental additional function: they control the crystallization process of the lipid phase. Furthermore, the emulsifier might modify the crystallization kinetics and natural polymorphic habit of the raw lipid materials used, avoiding recrystallization and destabilization problems of lipid nanoparticles during storage and application. Thus, in some systems, an appropriate combination of surfactants can facilitate the formation and stability of SLNs and NLCs [33,45]. Although bioactive ingredients are better solubilized in liquid lipid, the mixture of lipids in NLCs causes slower polymorphic transition and low crystallinity index [44].

In summary, the development of NCLs with different lipid materials can be used in a wide range of applications, showing higher encapsulation efficiency and more bioavailability, when compared to SLCs. Thus, lipid nanoparticles are promising bioactive compounds delivery systems (i.e., antimicrobials) for the formulation of ingredients in the industry.

## 4. Encapsulation of Natural Antimicrobials into Lipid-Based Nanostructures

As mentioned above, the growing consumer demand for more healthy and safe products has raised the interest for novel and natural antimicrobial agents as strategies to eliminate or inhibit microbial growth, avoiding the consumption of synthetic preservatives. Natural antimicrobials are used in the food and cosmetic industry in different products to ensure microbiological safety, while the pharmaceutic industry researches their potential for the development of new drugs. These natural antimicrobial agents have demonstrated diverse activity mechanisms against bacteria, including pore-forming in membranes, inhibiting cell-wall synthesis, altering cytoplasmic membrane, binding nucleic acids, inhibiting nucleic-acid synthesis, producing reactive oxygen species, and interfering with nutrient assimilation [59,60,61]. However, some drawbacks have been particularly associated with the direct incorporation of natural antimicrobials in foods, including the partial loss of antimicrobial activity due to cross reactions with the food components, and also, deterioration during thermal processing steps and storage [62]. In addition, some antimicrobials compounds, e.g., essential oils, present low water solubility, low physicochemical stability, and also may provide negative sensorial properties to the foods, affecting the flavor, color and texture [62].

**Figure 3:**
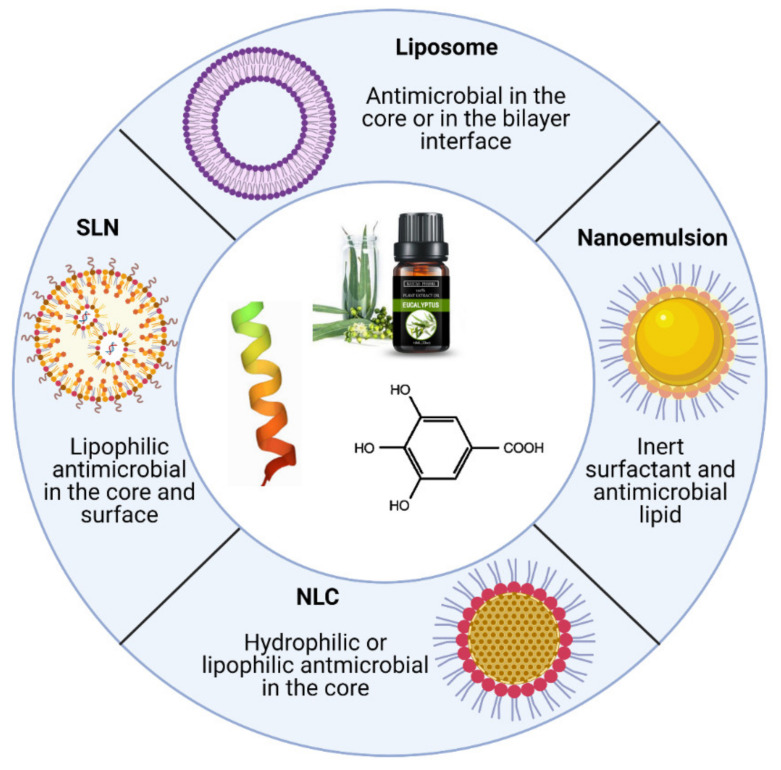
Lipid-based nanocarriers used as delivery systems for natural antimicrobials.

Lipid-based nanoparticles are one of the most promising nanoencapsulation systems in the food industry because of their favorable properties, including the capacity to encapsulate hydrophilic and lipophilic compounds in the same structure, targetability, production with food-grade materials, and application in diverse products without undesirable effects [63]. Another advantage of the encapsulation in lipid nanostructures is the cytotoxicity reduction of the antimicrobials as compared with their free form, as some reports describe the reduction of toxicity of antimicrobial peptides loaded in liposomes [64], essential oils loaded in NLC [65] and natural extracts encapsulated in NLC [66]. The encapsulation of antibacterial agents, in addition to the direct effect on bacterial cells, has also been reported to be effective against biofilms, preventing their formation on surfaces and destroying adhered films [67], and could play an outstanding role in the reduction of foodborne diseases, particularly those caused by antibiotic-resistant bacteria. The diversity of lipid-based nanoparticles is another advantage which facilitates their use in different food formulations, as shown in the Figure 3. The selection of a specific deliver systems for antimicrobials requires the consideration of several parameters including: the load capacity, toxicity, possible interactions with other components of the food matrix, the physical and chemical stability (size, zeta potential, polydispersity index), scalability, and cost. The recent research has demonstrated that lipid-based nanocarriers are promising tools for deliver natural antimicrobial compounds in foods, as will be shown in the next sections.

### 4.1. Encapsulation of Antimicrobial Peptides and Proteins

Antimicrobial peptides (AMPs) are small sized peptides of between 10 and 60 amino acids that can be charged (cationic or anionic) or not. The subclass of cationic AMPs presents positive charge, owing to the presence of large number of lysine or arginine residues in the amino acid primary backbone. Due to their positive charge, they easily interact with the microbial membranes which are negatively charged; however, the antimicrobial mechanism of cationic AMPs is not completely understood [68]. These compounds have several useful applications as antibacterial, antiviral and antifungal [69]. Among the diverse natural sources of AMP, including fungi, amphibians, insects, mammals and even chemically synthesized peptides, the peptides produced by bacteria (bacteriocins) are the most studied for application as food preservatives [70]. Bacteriocins such as nisin, subtilosin, pediocins, and lactacins are AMPs produced by various bacterial species such as Lactic Acid Bacteria (LABs) and *Bacillus* spp. These substances have demonstrated potential applications in preservation of food products against pathogenic and spoilage microorganisms [71].

Engineered lipid-based formulations entrapping AMPs and enzymes have been used as a strategy to improve the stability, maintaining the antimicrobial activity and providing a controlled release effect; some examples include liposomes [29,72], SLNs [73] and nanomicelles [74]. According to the current research, the use of lipid-based structures for nanoencapsulation of AMPs and enzymes presents two main advantages: first, providing protection from enzymatic and chemical degradation, maintaining the AMPs bioactive properties and, in the case of antimicrobial enzymes, preserving its inherent enzyme affinity to the substrate molecules; second, the carriers could increase the efficacy and availability, enabling a controlled and targeted delivery.

Some of the studies related to use of lipid-based carriers for AMPs and antimicrobial enzymes published in the last 10 years are summarized in Table 2.

The information compiled in Table 2 indicates that liposomes are the structures most frequently reported as nanocarriers for AMPs and enzymes. Liposomes are probably the most studied lipids carriers for bioactive compounds, and since the first approved pharmaceutical liposomal formulation of doxorubicin (Doxil) in the year 1995 by the U.S. Food and Drug Administration (FDA), a high number of other drug-containing liposomes have been approved by the European Medicines Agency (EMA) and the FDA for a wide range of pharmaceutical applications such as formulations to combat fungal diseases, cancer therapy and vaccines. These facts, summarizing the inherent liposome properties, have encouraged food scientists to develop liposomal carriers for AMPs and enzymes for food safety proposes. However, the use of others structures such as SLNs [73], nanomicelles [74] and nanoemulsions containing peptide antimicrobials [91] were also reported as innovative strategies for enhancing the activity of AMPs for food applications.

As it can be observed in Table 2, the bacteriocin nisin is the most studied AMP for lipid-based nanoencapsulation in the field of food science. This bacteriocin has received attention because of its activity against a variety of Gram-positive bacteria, including the foodborne pathogens *L. monocytogenes* and *S. aureus* [93]. Nisin is a food additive (E234) approved by the European Food Safety Association (EFSA) and Generally Recognized as Safe (GRAS) by the US Food and Drug Administration (FDA) [94]. Over the last 10 years of research on liposome encapsulation of nisin, it has been reported that this method improves nisin stability when used in complex food systems [22], retaining antibacterial and antibiofilm activity [84] and also providing controlled release effect [22,86]. This stability effect was unveiled in a recent work [43], where using scanning calorimetry (DSC) and small angle X-ray scattering (SAXS), it was demonstrated that the presence of nisin promoted the transition of lamellar to the cubic phase of phosphatidylcholine (PC) liposomes, leading to the formation of partial cubosomes dispersions, which presented better stability as compared with the unloaded liposomes.

Liposomes were also used as carriers for other bacteriocins with potential as food biopreservatives. One of the most studied is pediocin, an AMP produced by *Pediococcus acidilactici* with antilisterial activity [95]. Similar to the results with nisin, nanoliposomes containing pediocin have shown an improved antilisterial activity, stability and controlled release of pediocin [76,77,78]. Recently, the liposome encapsulation of the bacteriocin CAMT2 produced by *Bacillus amyloliquefaciens* ZJHD3-06 was reported [79], the liposomal formulation demonstrated low impact on the functional structure and crystallinity of bacteriocin, maintaining the antilisterial activity and stability when used in skim milk under refrigeration. In the same way, the antimicrobial peptide P34, obtained from the strain *Bacillus* sp. P34, was encapsulated in PC liposomes, resulting in similar antimicrobial activity against *L. monocytogenes* as compared to the free peptide [72]. In another work, the encapsulation of the AMP MccJ25 into liposomes coated with pectin and whey proteins was studied [80]; the authors observed that the two coatings improved the efficiency of encapsulation (EE) and promoted less degradation of the MccJ25 bacteriocin during simulated digestion as compared with the liposomes with one coating or without coating.

Compared with studies that concern bacteriocins, there are fewer works that explore the properties of AMPs obtained from animal and vegetal sources, where the preferred lipid carrier is also the liposomes. In this context, the development of liposome carriers for the antilisterial peptides Alpep10 and Alpep7, derived from anchovies and rice bran protein, respectively, has been described [82,83]. The liposomal formulations presented increased antibacterial and anti-biofilm formation activities against *L. monocytogenes*.

In our search for reports of lipid-based encapsulation of antimicrobial enzymes, only a few examples using liposomes were found. Indeed, there are not a great number of enzymes which can be used as food preservatives. Some works are related to the liposome encapsulation lysozyme, a food preservative recognized as safe which has antibacterial activity against Gram-positive and Gram-negative bacteria [96]. The development of PC-cholesterol liposomes containing lysozyme and the evaluation of its stability in simulated gastrointestinal fluid (SGF) and simulated intestinal fluid (SIF) was reported [87]; the best formulation presented an EE of 75.36%, low release of lysozyme in SFG (25% in 4 h) and acceptable stability in SIF (release of 40% in 4 h). Additionally, the encapsulation of lysozyme into PC-liposomes covered by polysaccharides was also reported in [29]; the results showed that the free and liposome loaded lysozyme presented 100% of antimicrobial activity against *L. monocytogenes*, however, only the free enzyme showed activity against *S. enterica* serotype Enteritidis. This result was attributed to the low release and a possible partial denaturation of the enzyme during liposome production. In addition, more recently, some researchers have explored the use of endolysins, as a novel approach of antimicrobial enzyme-based treatments for application in the food sector. These bacteriophage-encoded enzymes degrade specifically bacterial peptidoglycans and are active essentially against Gram-positive bacteria [97]. This approach, despite the low encapsulation efficiency (35.27%) obtained in the encapsulation of the endolysin BSP16Lys [88] and the reduced anti-biofilm activity of the endolysin LysRODI [89], is a promissory alternative for novel antimicrobial enzymes in the food industry.

As observed here, lipid-based nanocarriers are promising tools to improve the functional role of AMPs and provide more opportunities for their use in the food industry. However, there are still challenges regarding the AMPs incorporated in lipid-based delivery systems, such as the low efficiency of encapsulation, finding the equilibrium between the controlled release and effective antimicrobial activity, and also, understanding how these structures interact with a complex food matrix.

### 4.2. Encapsulation of Essential Oils

Essential oils (EOs) are natural, aromatic oily liquids with complex compositions that exhibit important biological activities, such as antifungal, antioxidant, and bactericidal. They are formed by aromatic plants as secondary metabolites, and are constituted mainly by a mixture of esters, aldehydes, ketones, terpenes, and phenolic compounds; for this reason, EOs have many applications in food, pharmaceutical and cosmetic industries [98]. However, because of EOs’ reactivity and hydrophobicity, their incorporation in water-based food and beverage products requires the stabilization of EOs by a suitable carrier [99]. Conventionally, encapsulation of EOs has represented a viable approach to ensure the physical stability of EOs, including their protection from evaporation, and to enable their controlled release. In the last years, different encapsulation systems for EOs have been exhaustively reviewed [100,101,102,103]. In particular, the recent literature reports many different formulations of EO nanoemulsions as natural food preservatives. Emulsions are biphasic, nano-sized dispersion of two immiscible liquids (mostly water and oil), which could be either water-in-oil (W/O) or oil-in-water (O/W) droplets, stabilized by an amphiphilic surfactant or emulsifier, and have been reviewed by different authors [99,103,104]. However, due to the liquid state of the disperse phase, where the payload is contained, the release rate is faster compared to systems with a solid or mesophase core [103], indicating that SLNs and liposomes have some advantages over nanoemulsions, due to their tunable composition, surface charge, and slower release and leakage rate; for this reason, in the present work we will focus on some works of SLN and liposomes for the encapsulation of EOs. Some examples are presented in Table 3.

In the case of EOs encapsulation in SLNs and NLCs, depending on the EO concentrations, significant alterations can be obtained in the core lipid crystalline structure which define the type of structure and functionality. For example, SLNs prepared by the addition of 0.03% w/v of *Zataria multiflora* Boiss. EO in the lipid matrix (5% w/v), were developed to use in the control of fungal pathogens, obtaining higher in vitro antifungal efficacy as compared with the free EO [105]. On the other hand, NLCs were fabricated and mixed at the 50:50 weight ratio with the lipid phase, for delivery of *Ridolfia segetum* Moris EO, where the EO acted simultaneously as a nanostructuring component of the NLC and as the payload bioactive compound [65]. In addition, the encapsulation in SLN or NLC might cause degradation of thermolabile EO components due to the preparation temperatures (around 70 °C). However, it was reported that citral-loaded NLCs, produced by the lipids Miglyol^®^, Poloxamer^®^ and Precirol^®^ melted at 70 °C and mixed with the EO presented high stability, sustained release and lower values of MIC against *E. coli*, *S. aureus* and *Bacillus cereus*, as compared with a nanoemulsion of citral [106]. Therefore, innovative formulations for the encapsulation of EOs in NLCs or SLNs could be a promising strategy to improve their antimicrobial efficiency offering controlled release and physicochemical stability.

Another alternative for lipid-based carriers for EOs is liposomes. Due to their mesophase structure, the EO release from liposomes occurs at an intermediate rate between nanoemulsions and SLNs, in addition, the EOs decreases the size of liposomes and reduces the oxidation and fluidity of the lipid bilayer [103]. Thereby, physicochemical properties of liposomes such as the size and EE can be affected by several parameters such as the composition of the lipid membrane, the preparation method and the kind of EO [112]. In this regard, recently it was reported that the liposome encapsulation of some EOs (estragole, isoeugenol, eucalyptol, terpineol, pulegone, and thymol) by the ethanol injection method improved their solubility and chemical stability [107]. In addition, the liposome encapsulation of salvia oil (SO) was performed in order to increase its stability and antibacterial effect on *S. aureus* biofilm [108]; as a result, the SO nanoliposomes showed prolonged delivery of SO and long-term anti-biofilm activity in milk containers, demonstrating that EO encapsulation into liposomes may have a place in the fight against foodborne pathogens.

In this context, the use of chrysanthemum EO (CHEO) loaded in liposomes to inhibit the growth of *Campylobacter jejuni* on chicken was reported [109]. The (CHEO) liposomes were modified with chitosan and pectin using a layer-by-layer electrostatic deposition method to obtain a triple-layer CHEO-liposomes; this formulation showed high antibacterial activity against *C. jejuni* on chicken during 14-days storage at a temperature range (4–37 °C) with no impact on surface color and sensory evaluation of chicken. Similarly, solid liposomes containing clove oil were studied as an alternative to remove *E. coli* O157:H7 biofilm from vegetables [110], obtaining as a result optimal activity at 4 and 25 °C without any change in flavor and quality of vegetables. In another recent work, the ability of nutmeg essential oil (NEO) loaded into solid liposomes (NEO-SLP) to preserve the quality and shelf life of pork meat batters was studied [111]. According to the authors, the addition of NEO-SLP was able to protect the meat batters during storage, reducing the microbial contamination and lipid oxidation. However, despite the different advantages such as increased stability, antimicrobial activity and reduction of the sensorial effect of EOs by liposome encapsulation, some limitations still persist for their large-scale production, including, low loading capability, high cost of materials and the need for complex preparation procedures.

### 4.3. Encapsulation of Plant Extracts

In addition to AMPs and EOs, other plant-derived antimicrobials are gaining interest from consumers and researchers for their uses as alternatives to the synthetic antimicrobial agents used as food preservatives. Phytochemicals are a wide group of chemical compounds including alkaloids, sulfur-containing phytochemicals, terpenoids, and polyphenols, with antioxidant and antimicrobial activity [113]. Nonetheless, due to their complex composition there are many challenges for the application of these kinds of natural antimicrobials in foods, such as low solubility, volatility, degradation during the process, and adverse effects on sensory properties of the food [114]. These limitations may be overcome using nanoencapsulation in lipid-based carriers instead of applying the free antimicrobials directly into the food. These platforms possess biocompatibility, biodegradability and are considered as a promising system for antimicrobial phytochemicals. In this section we will present a sample of the recently published works on the use of lipid-based structures as carriers for antimicrobials such as sulfur-containing phytochemicals and phenolic compounds. Some recent studies regarding lipid-based carriers for phytochemicals for food applications have been summarized in the Table 4.

As observed, the main bioactive compounds explored for lipid-based encapsulation are plant extracts rich in polyphenols. Besides their well-recognized antioxidant activity, some phenolic compounds exhibit high antibacterial activity, representing an important and promissory study field for the development of natural food preservatives in food [124]. However, due to the existence of unsaturated bonds in the molecular structures, phenolic compounds are very sensitive to oxidizing environments, such as light, oxygen, and moisture among others, which decreases their bioactive properties. Thus, the effectiveness of phenolic compounds depends on the preservation of their stability and bioactivity, and possible enhancement of their bioavailability, which can be achieved with encapsulation [125].

The encapsulation of natural antimicrobial phytochemicals for food applications into SLNS and NLCs has not been notably developed, which can be attributed to the newer appearance and exploration of these structures compared to the liposomes in the food science area. Some works related to the use of NLCs for encapsulation of plant extracts have reported the improvement of turmeric extract antimicrobial activity against *E. coli* [115], increased green tea stability [55] and reduction of toxicity of some antimicrobial natural compounds on human cultured cells after NLC encapsulation [66]. Furthermore, some recent research uses both structures, liposomes and NLC, for encapsulation of the same antimicrobial extract, and this offers an interesting opportunity to analyze the properties of each approach. Turmeric extracts loaded in liposomes (TNL) and NLC (T-NLC) were reported in [115,122]; in these works, both nanostructures showed similar size (around 100 nm) and particle size distribution (around 0.4), high EE (95 and 98%) and also better antimicrobial activity as compared with the free turmeric extract; however, the T-NLC presented higher values of MIC against *E. coli* (18.5 mg/mL) and *S. aureus* (0.079 mg/mL), as compared with the TNL (0.58 mg/mL and 0.07, respectively). This difference could be related to the behavior and velocity of the bioactive release of each lipid-based structure, which in some conditions tend to be lower in NLC [66].

In regard to phytochemicals encapsulation in lipid carriers, the liposomes continue to be the most studied approach, as mentioned above for AMPs and EOs. Recent reports of liposome encapsulation plant extracts explored the direct addition in food stuffs, as antimicrobial an antioxidant additive. As an example, recently the development of nanoliposomes of pistachio green hull extract was reported and tested as an additive in mayonnaise; as a result, the liposomes loaded with phenolic compounds (1 mg/g) showed high inhibitory efficiency on total viable fungal counts and identical effect on *Enterobacteriaceae* and lactic acid bacteria when compared with the free extract [63]. More recently, the use of nanoliposomes as carriers for *Laurus nobilis* leaf extract was reported, obtaining a liposome formulation with an encapsulation efficiency of 73.7 % of phenolic compounds; this formulation showed inhibition of *E. coli* and *S. aureus*, reduction of microbial spoilage and oxidation of minced beef [116]. In another work, the liposomes content of 1% and 2% of ethanolic coconut husk extract (ECHE) with a high content of phenolic compounds (370 mg tannic acid equivalent/g) was developed and characterized, where the liposomes content ECHE presented better antioxidant and antimicrobial activities compared with the free extract, and according to the authors, this result was due to the exposition of hydroxyl groups of the ECHE in the liposomes and the electrostatic liposome–bacteria cells interactions, which favored both bioactive properties [117]. Unfortunately, the chemical complexity of natural extracts and their changes during the encapsulation process make it difficult to understand their interactions with the lipid bilayer of liposomes and to clearly determine the mechanism that results in the increase of antimicrobial and antioxidant activities observed in the works described above. However, it seems that the liposome encapsulation of these extracts increases their potential as natural food preservatives with antioxidant and antimicrobial properties.

Another important group of natural antimicrobial compounds are the sulfur-containing phytochemicals, such as those present in garlic. Currently, the garlic (*Allium sativum L*.) organosulfur compounds, also called thiosulfinates, are under constant research due to its properties such as anticancer, antimicrobial, antioxidant, anti-inflammatory and antihypertensive [126]. However, the allicin (diallyl thiosulfinate), which represents 70–80% of the garlic sulfur compounds and possesses activities against a broad range of Gram-positive and Gram-negative bacteria, is volatile, unstable and susceptible to degradation [127]. In this context, liposomes encapsulation was studied as a strategy to preserve the thiosulfinates antimicrobial properties. Phosphatidylcholine (PC) liposomes loaded with an aqueous extract of garlic were developed and its antilisterial activity was tested in whole milk as a food model [121]. In this work, despite the low EE of 47.5% (of allicin) and its irregular morphology observed in the TEM analysis, free and encapsulate garlic extract (GE) showed similar bacteriostatic effect against different strains of *Listeria* spp., indicating the preservation of antilisterial properties after the liposome fabrication process, that includes heating at 55 °C and sonication. More recently, the liposome encapsulation of GE was also performed using PC and oleic acid (OA) as stabilizer [31]; the developed PC–OA–GE liposomes presented spherical morphology and EE of 79.7%; in addition, when compared with the free GE, the formulation of PC–OA–GE presented better results in situ used as a preservative against environmental molds in wheat bread due to its improved thermal properties, demonstrating great potential as an antifungal in baked food products.

Despite of the lack of information about the possible reduction of the taste of “garlic” after encapsulation, their application in cooked or baked foods looks promissory due to the high temperatures that could reduce the sensorial effect of the thermal-sensitive thiosulfinates. Therefore, the results of the works indicate that liposomes constitute a suitable system for encapsulation of the garlic sensitive compounds, increasing their stability, and presenting different applications in the food industry.

### 4.4. Co-Encapsulation of Natural Antimicrobials: Improving the Antimicrobial Efficacy?

**Figure 4:**
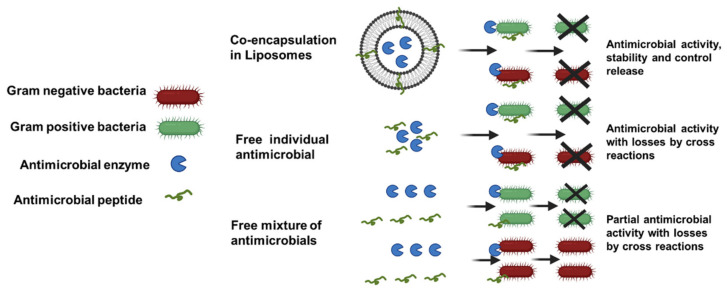
Schematic representation of the antibacterial synergetic effect of free and co-encapsulated antimicrobials.

Combining antibacterial agents is a strategy that may enhance the efficacies and spectrum of currently available antimicrobials for the inhibition of pathogenic and spoilage microorganism, due to the activity of different compounds with different antimicrobial mechanisms at the same time. This approach takes advantage of the synergistic effects of different antimicrobial compounds that can help to decrease the resistance of bacteria using lower doses of both agents [128]. However, despite the effectiveness of this technique as demonstrated by several researchers, studies using the co-encapsulation of antimicrobials in nanostructures are scarce. In this regard, co-encapsulation in lipid-based nanostructures such as liposomes has proved to be especially useful, due to other different advantages, such as the possibility of hydrophilic and lipophilic compounds being entrapped at the same time, controlled release, biocompatibility and protection of undesirable reactions with the food matrix. Some examples of this approach include the liposome co-encapsulation of AMPs with enzymes [29] and AMPs with plant extracts [129], where the synergetic effect of two different antimicrobial compounds against Gram-positive and Gram-negative bacteria was observed, as represented in the Figure 4, including the advantages of encapsulation such as protection and controlled release in a food matrix.

The liposome co-encapsulation of different mixtures of natural antimicrobials (plant extracts, lysozyme and nisin) was previously reported [130]. The produced liposomes presented variability in EE, stability and antimicrobial activity against Gram-positive and Gram-negative bacteria; the liposomes best results were obtained in the co-encapsulation of herbs and lysozymes, presenting high stability and the largest antimicrobial spectrum. More recently, the liposome co-encapsulation of lysozyme with nisin was reported [29]; in this approach, the prepared nanoliposomes containing the two antimicrobials were coated with polysaccharides (pectin and polygalacturonic acid) resulting in particles with high encapsulation efficiency (around 85%) for both antimicrobials. The free and encapsulated mixture of antimicrobials presented synergetic effects in the inhibition of *L. monocytogenes* and *Salmonella* Enteritidis; nevertheless, the lysozyme-nisin liposomes were more effective in the control the growth of *L. monocytogenes* in whole and skim milk stored at 7 °C (Figure 4). Commonly, *Salmonella* spp. and *E. coli* are resistant to nisin and other Gram-negative bacteria due to the architecture of its outer membrane, which prevents permeation of bacteriocins, acting as a barrier and conferring resistance [131]. In this example of the synergetic effect, the lysozyme hydrolyzes the peptidoglycan layer of the *Salmonella* cytoplasmic membrane favoring the antimicrobial effect of nisin on the Gram-negative bacteria, and this mechanism of activity is not affected by the liposome encapsulation, which also was able to reduce the negative effect of the milk fat on the antimicrobials [29]. The synergetic effect and improved activity in whole milk was also observed in our work of liposome co-encapsulation of garlic extract (GE) and nisin [129], where, the mixture of natural antimicrobials produced a significant decrease of bacterial *S. aureus*, *L. monocytogenes*, *E. coli* and *S.* Enteritidis, whereas free nisin and GE were not effective against any of these pathogens; also the GE-nisin liposomes showed continuous reduction of the viable counts of *L. monocytogenes* in whole milk under refrigeration (7 °C) for 25 days. As nisin alone was not effective against Gram-negative bacteria, the pore formation activity of allicin from the garlic extract [132] could have favored the permeation of nisin, making the Gram-negative bacteria more susceptible to the antimicrobial action.

Therefore, the co-encapsulation of antimicrobials in lipid-based nanocarriers is an innovative approach that makes it possible to preserve the functional activity of two or more natural antimicrobials with different characteristics of solubility, volatility and nature, enhancing their synergistic effect against foodborne pathogens. However, it is necessary to carry out more research with economically viable models to make it possible to develop new and more efficient conservatives for foods based in lipid-based nanocarriers.

## 5. Conclusions and Perspectives

The continuous progress in encapsulation technology and materials science has resulted in a variety of lipid-based delivery systems that provide effective transport and controlled release of antimicrobial substances. These nanostructures can be employed as excellent carriers for natural antimicrobial compounds, as the structures can be formulated by varying their composition, size, surface properties and membrane fluidity. Hydrophobic, hydrophilic, and amphiphilic compounds can be incorporated into lipid-based nanostructures, making these materials versatile carriers for antimicrobials. In fact, successful entrapment of antimicrobial peptides, enzymes, essential oils and antimicrobial phytochemicals into lipid nanostructures has been achieved by different researchers. Furthermore, different biopolymers, such as polysaccharides and proteins, can be included in the formulation of lipid nanostructures, resulting in improved characteristics of stability and release and compatibility in different delivery systems. Despite the fact that liposome encapsulation has been extensively investigated, further studies should be conducted to exploit the attractive characteristics of SNLs and NLCs as carriers of natural antimicrobials. Additional research for the development of lipid-based nanostructures that can be used synergistically with conventional preservation methods and/or that promote triggered antimicrobial release would also be useful to promote microbial safety in the food industry and other sectors.

## Author Contributions

Conceptualization, A.B; writing—original draft preparation, C.M.B.P. and N.A.L.; writing—review and editing, A.B. All authors have read and agreed to the published version of the manuscript.

## Figures and Tables

**Table 1:** Examples of some natural antimicrobials applied in food preservation.

Natural Antimicrobial	Microorganism Tested	Reference
Essential oils		
Grapefruit peel	*Salmonella parathypi* A, *Vibrio vulnificus* and *Seratia liquefaciens*	[14]
Oregano	*Escherichia coli* and *Staphylococcus aureus*	[15]
Thyme	*Escherichia coli* and *Bacillus subtilis*; *Clostridium perfringens*	[16,17]
Pink pepper	*Staphylococcus aureus*, *Bacillus subtilis*, *Listeria monocytogenes* and *Listeria innocua*	[18]
Plant extracts		
*Punica granatum*, *Syzygium aromaticum*, *Zingiber officinales* and *Thymus vulgaris*	*Bacillus cereus, Staphylococcus aureus, Escherichia coli, Pseudomonas aeruginosa* and *Salmonella typhi*	[19]
*Centella asiatica*	*Bacillus cereus, Escherichia coli* O157: H7, *Salmonella* enterica serovar Typhimurium, *Staphylococcus aureus, Aspergillus niger,* and *Candida albicans*	[20]
*Psidium guajava*, *Salvia officinalis*, *Ziziphusspina christi*, *Morusalba* L., and *Oleaeuropaea* L	*S. aureus*, *E. coli*, *Pasteurella multocida*, *B. cereus*, *Salmonella* Enteritidis and *M. gallisepticum*	[21]
Peptides and proteins		
Nisin	*Listeria monocytogenes* ATCC 7644, *L. monocytogenes* 4b, *Listeria* sp. str1, *L. innocua* 6a, and Listeria sp. str2	[22]
Pediocin	*Oenococcus oeni*	[23]
Lysozyme	*S. aureus* and *L. monocytogenes*	[24]

**Table 2:** Recent published articles about antimicrobial peptides loaded into lipid-based nanostructures.

Nanoparticle Type	AMP or Enzyme	Composition ^1^	Target Bacteria	Result of Encapsulation	Reference
Liquid crystal nanoparticle	Gramicidin A′, Melittin, Alamethicin, Cepropin A, Indolicidin and Pexiganan	Monoolein and phytantriol, with the addition of NaCl or DOPS	*Staphylococcus aureus, Bacillus cereus, Escherichia coli,* and *Pseudomonas aeruginosa*	Increased antimicrobial activity	[75]
Solid lipid nanoparticle	Nisin	Imwitor 900, poloxamer 188, sodium deoxycholate	*Listeria monocytogenes DMST 2871* and *Lactobacillus plantarum TISTR 850*	Extended antimicrobial activity by 20 and 15 days	[73]
Liposome	Pediocin	Soybean PC	*Listeria innocua*	Increased antimicrobial activity	[76]
Liposome	Pediocin	Soy lecithin, Soybean PC	*L. innocua*	Increased antimicrobial activity	[77]
Liposome	Pediocin	Partially purified PC	*L. monocytogenes, L. innocua* and *L. ivanovii*	Similar antimicrobial activity to the free pediocin	[78]
Liposome	Bacteriocin CAMT2	Soybean PC	*L. monocytogenes* ATCC 19111	Increased antimicrobial activity in whole milk	[79]
Liposome	Bacteriocin MccJ25	DMPC, DMPG, DMTAP, WPI, pectin	*Salmonella enterica* serotype Enteritidis	Reduced antimicrobial activity	[80]
Liposome	Sakacin 2a	Soybean PC, DOTAP	*L. monocytogenes* Scott A	Similar antimicrobial activity to the free Sakacin 2a	[81]
Liposome	Peptide P34	SoybeanPC	*L. monocytogenes* ATCC 7644	Reduced antimicrobial activity	[72]
Liposome	AMP Alpep10	DPPC, DMPG, cholesterol and chitosan	*L. monocytogenes*	Antibacterial and anti-biofilm activities	[82]
Liposome	AMP Alpep10	PPC, stearylamine, cholesterol	*L. monocytogenes*	Anti-biofilm activity	[83]
Liposome	Gramicidin	DODAB	*E. coli* and *S. aureus*	Increased antimicrobial activity spectrum	[64]
Liposome	Nisin Z	Soy lecithin, rhamnolipids	*L. monocytogenes, S. aureus, E. coli* and *P. aeruginosa*	Increased antimicrobial activity	[84]
Liposome	Nisin	Soybean PC, pectin and polygalacturonic acid	*L. monocytogenes* ATCC 7644	Increased antimicrobial activity	[22]
Liposome	Nisin	DOPC and DOPG	*L. monocytogenes*	Similar antimicrobial activity to the free nisin	[85]
Liposome	Nisin	Soybean PC, chitosan	*L. monocytogenes ATCC 7644, Listeria sp. str1, L. innocua 6a,* and *L. monocytogenes 4b*	Similar antimicrobial activity to the free nisin	[86]
Liposome	Lysozyme	PC and cholesterol		Some stability in SGF and SIF	[87]
Liposome	Lysozyme and nisin	PC and pectin	*L. monocytogenes* and *S. enterica* serotype Enteritidis	Increased antibacterial activity in milk	[29]
Liposome	Lysozyme and endolysin BSP16Lys	DPPC, cholesterol and hexadecylamine	*S. enterica* serotype Typhimurium *and E. coli*	Increased antimicrobial activity	[88]
Liposome	Endolysin LysRODI	Pronanosomes–pH	*S. aureus*	Similar antimicrobial activity to the free LysROD	[89]
Nanomicelle	Nisin	Monolaurin	*S. aureus*	Increased antimicrobial activity	[74]
Nano niosome	Nisin	Spam 80, sodium stearoyl lactate, and polyethylene glycol (PEG)	*S. aureus* and *E. coli*	Reduced antimicrobial activity	[90]
Nanoemulsion	Nisin and D-limonene	Stearic acid, sucrose stearate 170, and peanut oil	*S. aureus* ATCC6538, *Bacillus subtilis* ATCC6633 and *E. coli* ATCC8739	Increased antimicrobial activity	[91]
Nanoemulsion	ε-polylysine and D-limonene	Tween 80 and water	*E. coli, S. aureus, Bacillus subtilis* and *Saccharomyces cerevisiae*	Increased antimicrobial activity	[92]

^1^ DOPS, dioleylphosphatidylserine; PC, phosphatidylcholine; DMPC, dimiristoylphosphatidylcholine; DMPG, dimiristoylphosphatidylglycerol; DMTAP, dimiristoyltrimethylammonium propane, WPI, whey protein isolate; DOTAP, dioleyltrimethylammonium propane; DPPC, dipalmitoylphosphatidylcholine; PPC, polyunsaturated phosphatidylcholine; DODAB, dioctadecyldimethylammonium bromide; DOPC, dioleylphosphatidylcholine; DOPG, dioleylphosphatidylglycerol.

**Table 3:** Recent works of encapsulation of EOs in lipid-based nanocarriers.

Nanoparticle Type	Plant-Based Antimicrobial	Composition	Target Microorganism	Result of Encapsulation	Reference
NLC	*Zataria multiflora* essential oil	Glyceryl mono stearate, Precirol ATO and Polysorbate 80	*A. ochraceu, A. niger, A. flavus, A. solan, R. solani*, and *Rh. stolonifer*	Increased antifungal activity	[105]
NLC	*Ridolfia Ssegetum* (L.) Moris essential oil	Precirol ATO 5 and Polysorbate 80		Sustained dermal delivery profile	[65]
NLC	Citral	Miglyol, Precirol, Poloxamer and Polysorbate 80	*S. aureus*, *B. cereus*, *E. coli*, and *Candida albicans*	Reduction of antimicrobial activity	[106]
Liposome	Estragole, isoeugenol, eucalyptol, terpineol, pulegone, and thymol	Lipoid S100		Stability after long term storage at 4 °C	[107]
Liposome	Salvia oil	Soy lecithin and cholesterol	*S. aureus*	Prolonged antibiofilm activity	[108]
Liposome	Chrysanthemum essential oil	Soy lecithin and cholesterol	*Campylobacter jejuni*	Increased antimicrobial activity	[109]
Liposome	Clove oil	Soy lecithin and cholesterol	*Escherichia coli* O157:H7 biofilm	Increased antimicrobial activity	[110]
Liposome	Nutmeg (*Myristica fragrans Houtt*) essential oil	Soy lecithin and cholesterol		Improved the application in meat batters	[111]

**Table 4:** Recent works of encapsulation of antimicrobial phytochemicals in lipid-based nanocarriers.

Nanoparticle Type	Plant-Based Antimicrobial	Composition ^1^	Target Microorganism	Result of Encapsulation	Reference
NLC	Tumeric extract	Campritol 888-ATO, Miglyol 812 and poloxamer 407	*E. coli, S. aureus, Bacillus cereus, P. aeruginosa, Streptococcus mutans* and *Candida fungus*	Increased antimicrobial activity	[115]
NLC	Plumbagin, hydroquinone, eugenol, α-asarone and α-Tocopherol	PEO, PPO, poloxamer 188, Miglyol 812 N, Tristearin and polysorbate 80	*Clavibacter michiganensis* ATCC 27822, *Pseudomonas syringae* ATCC 19310, *Agrobacterium tumefaciens* DSM 30207, *Agrobacterium vitis* DSM 6383	Increased antimicrobial activity	[66]
NLC	Green tea extract	n-Hexadecyl palmitate, glycerol stearate, grape seed oil, Synperonic F68 and Tween 20	*E. coli* K12-MG1655	Increased antimicrobial activity	[55]
Liposome	*Laurus nobilis* leaf extract	Tween 80 and lecithin	*E. coli, S. aureus*	Increased antimicrobial activity	[116]
Liposome	Cocconut husk extract	PC and cholesterol	*S. aureus, E. coli, Vibrio parahaemolyticus, L. monocytogenes*, and *P. aeruginosa*	Increased antimicrobial activity	[117]
Liposome	Pistachio green hull extract	Lecithin	*S. aureus*, *Enterobacteriaceae*, molds and yeasts	Increased antimicrobial activity	[63]
Liposome	Cinnamaldehyde	PDA-NHS and DMPC	*E. coli W1485* and *B. cereus ATCC 14579*	Increased antimicrobial activity	[118]
Liposome	Cinnamaldehyde	Lecithin and α-tocopherol	*A. hydrophila, V. vulnificus, V. parahaemolyticus*, *V. alginolyticus*, *S. agalactiae*	enhanced survival rate and inhibits bacterial growth in zebrafish	[119]
Liposome	Limonene	PDA-NHS and DMPC	*E. coli*, *L. monocytogenes,* yeasts and molds	Increased antimicrobial activity	[120]
Liposome	Garlic extract	PC and oleic acid	Environmental molds	Increased antifungal activity	[31]
Liposome	Garlic extract	PC	*Listeria* spp.	Similar antimicrobial activity to the free garlic extract	[121]
Liposome	Turmeric extract	PC	*E. coli, S. aureus*, *B. cereus, P. aeruginosa*, *Streptococcus mutans* and *Candida albicans*	Increased antimicrobial activity	[122]
Liposome	Green tea extract	Lecithin, cholesterol, DSPE, PEG 2000	*B. cereus, S. enterica* serotype Typhimurium, *E. coli* O157:H7, *L. monocytogenes*	Increased antimicrobial activity	[123]

^1^ PC, phosphatidylcholine; DMPC, dimiristoyl phosphatidylcholine; DSPE, distearoyl phosphatidylethanolamine.

## Data Availability

No new data were created or analyzed in this study. Data sharing is not applicable to this article.
